# Supplementary material for: Blockchain for video watermarking: An enhanced copyright protection approach for video forensics based on perceptual hash function
Source: PLoS One. 2024 Oct 22;19(10):e0308451. doi: 10.1371/journal.pone.0308451 (PMC11495578; doi:10.1371/journal.pone.0308451)
Supplement: Attachment — Submitted filename: Reply-to-reviewers.docx [file pone.0308451.s001.docx]

To The Editor-in-chief and the Associate Editor,

**PLOS ONE**

Manuscript ID: **ONE-D-24-09209 R1**

Title:	**Blockchain for Video Watermarking: An Enhanced Copyright Protection Approach for Video Forensics based on Perceptual Hash Function**

Dear Sir,

Thanks for giving us the opportunity to revise the paper for the possible publication in your interesting journal. We also thank the reviewers for their constructive comments. We have significantly revised the manuscripts based the comments received and the details are summarized below.

**Reply to the comments:**

**In the revised version, what was modified and added was written in red**

**Reviewer 1**

1- **The paper provides innovative insights into using blockchain and perceptual hash functions for video watermarking.**

**Response**

Thank you for positive feedback

2- **It could benefit from a more detailed exploration of the limitations and challenges associated with this integration.**

**Response**

In the revised version, a new paragraph was added that explains in more detail the limitations and challenges associated with the integration of blockchain technology with digital watermarking and a perceptual hash function.

Integrating blockchain technology with digital watermarking and perceptual hash functions holds significant potential for enhancing content security, authenticity verification, and ownership tracking. However, this integration presents a range of limitations and challenges including:

-	Digital watermarks and perceptual hash functions generate data that needs to be stored or referenced on the blockchain. Storing large amounts of data directly on the blockchain can be expensive and impractical due to limited storage capacity and high transaction fees.

-	Blockchain networks, especially those with public and permissionless configurations, often face scalability issues. The processing speed of transactions might not be fast enough to handle real-time applications of digital watermarking.

-	The additional computational steps required for watermark embedding, hash generation, and blockchain transactions can introduce latency, which might not be acceptable in real-time applications.

-	Ensuring compliance with data privacy regulations is crucial when storing or referencing any personal or sensitive information on the blockchain. One potential solution is to store personal data off-chain and use the blockchain to store references or hashes of the data. This way, the actual data can be deleted or modified off-chain while maintaining the integrity and immutability of the blockchain records.

Subsection V LIMITATIONS AND CHALLENGES, Pages (37&38)

3- **The paper mentions the use of blockchain for storing watermark information but lacks detail on the specific blockchain architecture used (e.g., public vs. private blockchain) and how blockchain's inherent properties (e.g., immutability, decentralization) are leveraged to enhance copyright protection. Could the authors elaborate on the blockchain architecture and justify their choice in the context of video watermarking?**

**Response**

In the revised version, more sentences were added to specify in detail the blockchain architecture. Furthermore, the justification of our choice regarding blockchain used (public or private) and their type (immutability, or decentralization) was also mentioned.

“Using Truffle, Solidity, and IPFS within the suggested public blockchain framework provides a robust solution for video watermarking, ensuring transparency, security, and decentralized storage. This approach leverages the strengths of public blockchains to create immutable records of video ownership and authenticity while using IPFS for efficient and decentralized storage. IPFS allows for decentralized storage of large video files, which would be impractical to store directly on the blockchain. By storing the video on IPFS and recording the hash on the blockchain, you can ensure both decentralization and efficient access”.

“Our choice of public blockchain stems from the fact that public blockchains are decentralized and maintained by multiple nodes, making it nearly impossible to alter data once it's recorded. This immutability ensures that the ownership and authenticity of a watermarked video can be reliably verified by anyone, fostering trust among content creators, distributors, and consumers. Furthermore, public blockchains are maintained by a decentralized network of nodes, reducing the risk of data being controlled or manipulated by a single entity. This decentralization can enhance security and resilience against attacks or failures; this openness increases accountability and reduces disputes over content ownership”.

Page (13)

4- **Section III introduces a perceptual hash function for computing hash values based on the structural information of video frames. However, the computational complexity and scalability of this hash function are not discussed. Given the potentially large size of video files and the need for real-time processing in forensic applications, how do the authors address the computational demands of the perceptual hash function?**

**Response**

In the revised version, new paragraphs were added to discuss the scalability issue of utilizing the perceptual hash function for computing hash values based on the structural information of video frames. Furthermore, the computational complexity of building this type of hash was also highlighted.

“Using perceptual hash functions for video watermarking combined with blockchain technology can significantly enhance content verification and integrity. Addressing scalability challenges involves efficient hashing algorithms, segment-based hashing, and off-chain storage solutions like IPFS.  In our suggested model, we utilize a simplified version of pHash that can reduce computational overhead to generate compact hash values quickly. Furthermore, instead of generating perceptual hashes for every frame, divide the video into segments (e.g., every few seconds or key frames) and generate a hash for each segment. This reduces the number of hashes while still providing adequate verification. Finally, using IPFS to store the perceptual hashes and video files off-chain using decentralized storage solutions like IPFS. Only store the IPFS hash (a unique identifier) on the blockchain, which points to the actual data stored off-chain. By implementing these strategies, it is possible to achieve a scalable and robust system for managing and verifying video content in a decentralized manner”.

“The computational complexity of building perceptual hash functions for video watermarking is an important factor to consider, especially for scalability and real-time applications. This process generally depends on the specific algorithm used and the size of the video content. Its complexity is $O(n\log n)$ for the for the DCT computation, where $n$ is the number of pixels in the resized image/frame. So the total complexity is $O(F\times H)$, where $F$ is the number of frames and $H$ is the complexity of the perceptual hash function”.

Pages (17& 18)

5- **While the experimental section (Section IV) evaluates the approach against common video attacks, there is limited discussion on its resistance to more advanced manipulations, such as deep fake technology or sophisticated frame interpolation attacks. Can the authors provide insights or experimental evidence on the method's effectiveness against these emerging threats?**

**Response**

In the revised version, new paragraphs were added to highlight the suggested model’s resistance to more advanced manipulations, such as deepfake technology or sophisticated frame interpolation attack.

“Regarding the suggested model’s resistance to more advanced manipulations, such as deepfake technology or sophisticated frame interpolation attack. Deepfake algorithms primarily focus on the luminance channel to ensure high visual fidelity and realism in terms of shapes and edges [56]. In our suggested model, embedding watermarks in the chrominance channel can therefore survive these manipulations better than those in the luminance channel. Deepfake algorithms might not accurately replicate the subtleties of the chrominance channels, watermarks embedded in these channels can help in detecting inconsistencies and manipulations. By comparing the extracted watermark from a potentially deepfaked video with the original, it is possible to identify tampering. Also, as our model embeds the watermark in the frequency domain; it can make the watermark more resilient to deepfake manipulations [57].”

“Frame interpolation attacks involve generating intermediate frames between existing frames in a video to create smooth transitions, often using techniques like deep learning-based algorithms. This type of manipulation can distort or disrupt embedded watermarks. Our strategies for enhancing watermark robustness against frame interpolation rely on distributing the watermark information across multiple frames instead of embedding it in single frames. This way, even if some frames are interpolated or modified, the watermark can still be reconstructed from the remaining frames. Furthermore, adjust the watermark embedding process based on the content of each frame. This approach can use characteristics like motion vectors or scene changes to decide where and how to embed the watermark, making it more resilient to interpolation artifacts [58].”

Pages (33&34&35)

Furthermore, new sentences were added in the future work section for potential directions for further research and development of our work regarding its resilience to deepfake attacks.

“Currently, we haven't conducted a dedicated evaluation of the model's resistance against deepfakes. However, based on the watermark placement strategy, we believe the impact would be limited in most cases. Further investigation using deepfake datasets specifically designed to target watermarked content is necessary to fully assess the model's robustness in this scenario.”

Page (39)

6- **The integration of video watermarking with blockchain raises concerns about scalability and transaction throughput, especially when dealing with high volumes of video content. How does the proposed method address the scalability challenges associated with blockchain technology, and what are the implications for the performance and responsiveness of the copyright protection system?**

**Response**

In the revised version, new paragraphs were added that highlight how the suggested model addresses the scalability challenges associated with blockchain technology. Also, the implications for the performance and responsiveness of the copyright protection system were discussed in detail.

“The integration of video watermarking with blockchain offers a secure and tamper-proof solution for copyright protection. However, a major concern arises with scalability, particularly when dealing with large volumes of video content. The proposed method effectively addresses these challenges through IPFS storage and Merkle DAGs. Instead of storing the entire watermarked video directly on the blockchain, the system utilizes IPFS for efficient storage and retrieval of video data. IPFS's distributed storage architecture and content-based addressing mechanisms provide a scalable and cost-effective solution for handling large video files (off-chain watermark storage)”.

“Furthermore, to maintain data integrity while reducing blockchain storage requirements, the proposed method employs Merkle DAGs (directed acyclic graphs) to store hashes of the watermarked video content on the blockchain. Merkle DAGs allow for more efficient updates and verification of data compared to traditional Merkle trees. This is particularly beneficial for video watermarking, where new watermarks may be added or existing ones updated frequently. Additionally, Merkle DAGs are immutable, meaning once a hash is added, it cannot be altered. This ensures the integrity of the watermark data and prevents unauthorized modifications. Finally, Merkle DAGs provide greater flexibility in organizing and structuring data, making them well-suited for handling complex data relationships like those encountered in video watermarking”.

Page (24)

Yet, the use of blockchain for video watermarking in a copyright protection system can significantly influence both performance and responsiveness. Here are the key implications: (1) as more videos are watermarked and registered on the blockchain, the size of the blockchain grows. This can affect the speed of transaction validation and block creation. (2) Blockchain transactions, especially in public blockchains, can have a high latency due to the time required for block confirmation. This can delay the process of watermark registration and verification. (3) Blockchain's decentralized nature means that every transaction needs to be confirmed by the network, which can take time. This can affect the ability to perform real-time watermark verification. (4) The responsiveness of the system can vary based on the number and distribution of nodes. More nodes can mean more security but also potentially more latency.

By effectively utilizing IPFS and Merkle DAGs, the proposed method demonstrates a well-considered approach to addressing scalability challenges in blockchain-based video watermarking. The strategies employed were to minimize storage overhead, improve transaction throughput, and optimize performance for handling large volumes of video content. The use of Merkle DAGs in particular provides additional advantages in terms of immutability, efficiency, and flexibility, making it a suitable choice for securing and managing video watermark data in a scalable and secure manner.

Pages(27&28)

7- **Author can read the following papers to increase the technical strength of the paper:**

**Secure blockchain enabled Cyber–physical systems in healthcare using deep belief network with ResNet model**

**A Privacy-Preserving Authentic Healthcare Monitoring System Using Blockchain**

**Response**

Thank you very much for helping, the two paper were cited in the manuscript. Refs. [59]&[60]

**Reviewer 2**

1- **While the manuscript details experimental results showing memory efficiency and improved robustness due to the blockchain's random hash function, it could benefit from a more thorough comparison with existing methods. Specifically, the manuscript could include benchmark tests against traditional non-blockchain-based watermarking approaches to highlight the improvements quantitatively.**

**Response**

In the revised version, a new set of experiments was conducted to compare the suggested blockchain-based video watermarking technique with traditional non-blockchain-based watermarking approaches to highlight the improvements quantitatively.

The proposed blockchain–based technique has been compared with traditional two recent non-blockchain video-watermarking techniques presented in Refs. [61][62]. In Ref.[61], a lossless video watermarking system is suggested that operates by selecting keyframes optimally via the use of a linear wavelet transform and an intelligent gravitational search algorithm. Using the histogram difference approach, this methodology extracts color motion as well as static frames from the cover video. The chrominance channel of the motion frames undergoes a one-level linear wavelet transform, and a low-frequency sub-band LL is chosen for the watermark embedding. In Ref. [62], a secure video watermarking method is provided employing a novel two-dimensional complex map. In order to demonstrate the chaotic nature of the suggested map, standard analyzes have been done on a dynamical system. There is also an efficient approach for embedding and extraction watermark that uses single value decomposition in conjunction with the IWT, DWT, and CT transforms.

This set of experiments measures the imperceptibility performance of the proposed technique against existing techniques in terms of mean peak signal to-noise ratio (MPSNR) and Mean Structural Similarity Index Measure (MSSIM) under different video attacks. See [61] for more details regarding these metrics. Table 5 highlights the MPSNR and MSSIM values corresponding to each video against compared schemes. It can be noticed from the table that the proposed technique attains an average value of MPSNR as 48.89 and MSSIM value as 0.9998. These superior values confirm that the proposed technique outperforms the compared schemes. In general, Blockchain employs cryptographic techniques via hashing to ensure the security and privacy of data. Watermarking information stored on a blockchain can be encrypted and accessible only to authorized parties, reducing the risk of unauthorized access or manipulation.

Table 5: The imperceptibility (MPSNR, MSSIM) of the considered techniques under different attacks.

| Cover video | Our blockchain-based Technique | | Non-blockchain-watermarking Technique in Ref. [61] | | Non-blockchain-watermarking Technique in Ref. [62] | |
| --- | --- | --- | --- | --- | --- | --- |
|  | MPSNR | MSSIM | MPSNR | MSSIM | MPSNR | MSSIM |
| Silent | 48.98 | 0.9997 | 48.97 | 0.9995 | 42.65 | 0.9897 |
| Foreman | 48.05 | 0.9999 | 47.98 | 0.9997 | 42.57 | 0.9889 |
| Mobile | 49.34 | 0.9994 | 48.07 | 0.9993 | 41.98 | 0.9843 |
| Hall monitor | 48.87 | 0.9999 | 48.52 | 0.9999 | 42.37 | 0.9863 |
| **Average** | **48.89** | **0.9998** | **48.39** | **0.9996** | **42.39** | **0.9873** |

Pages (36&37)

2- **More detailed statistical analysis and error metrics could strengthen the validation of the experimental results.**

**Response**

The revised version strengthened the validation of the experimental results by measuring more statistical analysis and error metrics in the experiments.

In table 2, to determine robustness values, we used the mean normalized correlation (MNC), an essential metric for evaluating video watermarking methods. It measures the similarity between the original watermark and the extracted watermark. High values of MNC indicate that the watermark has been accurately extracted, implying robustness against various types of attacks or distortions.

In Table 1, The Average Angular Error (AAE) metric, which determines the average angle between the actual and estimated flow vectors, is used to assess the performance.

In terms of execution time (memory efficiency), the results of IPFS main procedure are described in Table 4.

Furthermore, both of MPSNR and MSSIM were used in Table 5 for comparative study

- Mean Peak Signal-to-Noise Ratio (MPSNR) is a metric used to evaluate the quality of watermarking techniques. It measures the fidelity of the watermarked image compared to the original image, taking into account both the peak signal-to-noise ratio (PSNR) and the mean squared error (MSE). MPSNR is particularly useful for assessing the robustness of watermarking algorithms against various attacks or transformations.

- MSSIM: Mean Structural Similarity Index (MSSIM) is another metric commonly used to evaluate the quality of watermarking techniques, especially in scenarios where perceptual fidelity is crucial. MSSIM assesses the similarity between the watermarked image and the original image by considering luminance, contrast, and structure.

3- **The manuscript mentions the system's resilience to video frame attacks but does not deeply explore potential vulnerabilities introduced by blockchain technology itself, such as scalability issues, 51% attacks, or other common blockchain weaknesses.**

**Response**

- In the revised version, new paragraphs were added that elaborate on how the suggested model handles 51% attacks or other common blockchain weaknesses.

“Handling a 51% attack on a blockchain used for video watermarking involves several strategies to mitigate the impact and prevent the attack. A 51% attack occurs when a single entity or a group of entities gains control of more than 50% of the network's mining or hashing power, allowing them to manipulate the blockchain. Here are some strategies used within the suggested model to handle and prevent such attacks within the context of video watermarking on a blockchain: (1) our blockchain network structure has a large and diverse number of nodes. The more nodes there are, the harder it is for any single entity to control the majority. (2) In our model, by using IPFS, the actual video content and watermarks are stored in a decentralized manner, making it difficult for any single entity to tamper with or control the data. Once data is stored on IPFS, it is immutable and can be verified using cryptographic hashes. While the perceptual hash is stored on the blockchain, the actual video data is stored on IPFS, which is a decentralized storage network. This reduces the impact of a 51% attack on the overall system. (3) The perceptual hash of a video (a compact representation of the video’s content) is small in size. Storing this hash on the blockchain instead of the entire video reduces the amount of data that needs to be processed and stored on-chain”.

Pages (34&35)

- Furthermore, in the revised version, new paragraphs were added that highlight how the suggested model addresses the scalability challenges associated with blockchain technology.

“The integration of video watermarking with blockchain offers a secure and tamper-proof solution for copyright protection. However, a major concern arises with scalability, particularly when dealing with large volumes of video content. The proposed method effectively addresses these challenges through IPFS storage and Merkle DAGs. Instead of storing the entire watermarked video directly on the blockchain, the system utilizes IPFS for efficient storage and retrieval of video data. IPFS's distributed storage architecture and content-based addressing mechanisms provide a scalable and cost-effective solution for handling large video files (off-chain watermark storage)”.

“Furthermore, to maintain data integrity while reducing blockchain storage requirements, the proposed method employs Merkle DAGs (directed acyclic graphs) to store hashes of the watermarked video content on the blockchain. Merkle DAGs allow for more efficient updates and verification of data compared to traditional Merkle trees. This is particularly beneficial for video watermarking, where new watermarks may be added or existing ones updated frequently. Additionally, Merkle DAGs are immutable, meaning once a hash is added, it cannot be altered. This ensures the integrity of the watermark data and prevents unauthorized modifications. Finally, Merkle DAGs provide greater flexibility in organizing and structuring data, making them well-suited for handling complex data relationships like those encountered in video watermarking”.

Page (24)

4- **Some assumptions are made regarding the blockchain's performance and security without detailed discussion or substantiation from real-world data or broader use cases.**

**Response**

In the revised version, new paragraphs were added that highlights in detail the assumptions that are made regarding blockchain’s performance and security.

“When considering blockchain's performance and security in the context of video watermarking applications, several assumptions are made, including: (1) Blockchain networks can face scalability issues when handling large volumes of data, such as video files and watermarks. (2) Blockchain's inherent design, which relies on consensus mechanisms, can introduce latency in transaction processing. (3) The transaction throughput of public blockchains is generally lower compared to centralized databases. (4) Storing large video files directly on the blockchain is impractical due to storage and cost constraints. (5) Blockchain networks are vulnerable to 51% attacks where an entity controls the majority of the network’s hash power or stake.

“To implement blockchain effectively in video watermarking, these assumptions guide several strategic decisions, including utilizing off-chain storage that stores large video files on decentralized storage solutions like IPFS while storing only hashes and metadata on the blockchain to ensure scalability and efficiency. Furthermore, using Solidity and Truffle, blockchain-based video watermarking systems can effectively address some of the inherent weaknesses of blockchain technology, providing a robust solution for protecting digital video content. Using Solidity, a smart contract is created to store the hash, ownership details, and licensing information on the blockchain. Truffle is used to deploy and manage this contract.

Pages (12&13)

5- **The manuscript could include more specific details about the implementation of the perceptual hash function and the blockchain structure. For example, the type of blockchain (public, private, and consortium) and the specific blockchain platform used could be specified to help replicate the study.**

**Response**

- In the revised version, more sentences were added to specify in detail the blockchain architecture. Furthermore, the justification of our choice regarding blockchain used (public or private) and their type (immutability, or decentralization) was also mentioned.

“Using Truffle, Solidity, and IPFS within the suggested public blockchain framework provides a robust solution for video watermarking, ensuring transparency, security, and decentralized storage. This approach leverages the strengths of public blockchains to create immutable records of video ownership and authenticity while using IPFS for efficient and decentralized storage. IPFS allows for decentralized storage of large video files, which would be impractical to store directly on the blockchain. By storing the video on IPFS and recording the hash on the blockchain, you can ensure both decentralization and efficient access”.

“Our choice of public blockchain stems from the fact that public blockchains are decentralized and maintained by multiple nodes, making it nearly impossible to alter data once it's recorded. This immutability ensures that the ownership and authenticity of a watermarked video can be reliably verified by anyone, fostering trust among content creators, distributors, and consumers. Furthermore, public blockchains are maintained by a decentralized network of nodes, reducing the risk of data being controlled or manipulated by a single entity. This decentralization can enhance security and resilience against attacks or failures; this openness increases accountability and reduces disputes over content ownership”.

Page (13)

- Furthermore, more paragraphs were added to specify in detail the blockchain platform used could be specified to help replicate the study.

In the context of IPFS, Bitswap is a crucial component responsible for efficiently exchanging blocks of data between peers in the network. Here's a detailed overview of how Bitswap operates within IPFS [50]:

(1) Block Retrieval: When a node in the IPFS network wants to retrieve a file or a specific block of data, it sends out a request to its connected peers.
(2) Wantlist: Along with the request, the node sends a "wantlist" to its peers. This wantlist contains entries for each block it currently desires but does not possess. Each entry typically includes the CID (Content IDentifier) of the desired block and the corresponding priority.
(3) Responses: Peers that receive the request check their own local storage to see if they have any of the requested blocks. If they do, they respond with those blocks.
(4) Block Prioritization: Upon receiving a request along with a wantlist, peers prioritize which blocks to send based on various factors including the urgency indicated in the wantlist and their own strategies for optimizing data exchange.
(5) Block Trading: After receiving blocks from peers, nodes may redistribute these blocks to other peers in need. This establishes a sort of block trading system where nodes help each other acquire the data they need.
(6) Duplicate Block Prevention: Bitswap includes mechanisms to prevent duplicate blocks from being sent unnecessarily. Peers keep track of which blocks they have already sent to specific peers, reducing redundant data transmission.
(7) Data Verification: Both sending and receiving nodes verify the integrity of the transmitted blocks using cryptographic hashes. This ensures that the received data hasn't been tampered with during transmission.
(8) Request Manager: Bitswap also includes a request manager component responsible for coordinating block requests and managing peer connections efficiently.
(9) Connection Management: Bitswap dynamically manages connections with peers in the network, establishing new connections as needed and optimizing the utilization of available network resources.

Pages (23&24)

- Also, more paragraphs were added for more specific details about the implementation of the perceptual hash function

Step 3: Perceptual Hash Function	Pages (17& 18&19)

- Finally, new paragraphs were added to highlight the blockchain platforms used, including Truffle and Solidity.

Solidity is a statically-typed programming language designed for developing smart contracts that run on the Ethereum Virtual Machine (EVM). Smart contracts are self-executing contracts with the terms of the agreement directly written into code. For blockchain-based video watermarking, Solidity can be used to develop smart contracts that manage various aspects of watermarking, including: (1) Ownership Verification: Smart contracts can be designed to store and verify the ownership of video content. When a video is uploaded, its unique watermark can be registered on the blockchain along with the owner's information. (2) Watermark Embedding and Extraction: While the actual embedding and extraction of watermarks are performed off-chain, smart contracts can be used to log these actions on the blockchain. For example, a hash of the watermarked video can be stored on-chain to verify its authenticity later. (3) Usage Tracking: Smart contracts can track how and when the video is accessed or used. Each time the video is played or shared, a record can be added to the blockchain, ensuring a transparent and immutable history of the video’s usage. (4) License Management: Smart contracts can manage the issuance and verification of licenses for video content. This ensures that only authorized users can access or distribute the video, reducing the risk of piracy.

Truffle is a development framework for Ethereum that provides tools to make writing and managing smart contracts easier. It includes features for compiling, deploying, and testing contracts, which are crucial for ensuring the reliability and security of blockchain applications. In the context of video watermarking, Truffle can help in the following ways: (1) Smart Contract Development: Truffle provides a structured environment for developing Solidity smart contracts. It includes libraries that simplify common tasks, such as interacting with the blockchain and managing contract state. (2) Automated Testing: Truffle allows for the creation of automated tests to ensure that smart contracts behave as expected. This is essential for verifying that the watermarking process is secure and that the contracts correctly handle ownership verification, usage tracking, and license management. (3) Deployment Management: Truffle simplifies the deployment of smart contracts to different blockchain networks. This includes managing migrations and ensuring that contracts are deployed in a consistent and reproducible manner. (4) Interaction with Frontend: Truffle integrates well with frontend technologies, enabling the development of user interfaces that interact with smart contracts. This is important for creating user-friendly applications for video content creators and consumers.

Pages (25&26)

6- **While the manuscript discusses the potential applications of the proposed system in video forensics, it could expand on the broader impact, including implications for copyright enforcement in digital media.**

**Response**

In the revised version, a new paragraph was added that highlights the potential implications of the suggested model for copyright enforcement in digital media.

There are other areas outside of video forensics that might benefit from using video watermarking solutions that are based on the blockchain and focus on digital media copyright enforcement, including (1) Enhanced Copyright Protection: Blockchain's immutable ledger can store detailed ownership information. Once a video is watermarked and registered on the blockchain, the ownership record cannot be altered or deleted, providing a clear and permanent record of who owns the rights to the content. (2) Streamlined Licensing and Royalties: Blockchain can automate licensing agreements through smart contracts. These self-executing contracts ensure that creators are automatically compensated when their content is used, reducing the complexity and cost associated with traditional licensing processes. (3) Traceability: Watermarked videos can be traced back to the source if pirated copies appear online. Blockchain can record every transaction and access point, making it easier to identify and take action against unauthorized distribution.

Page (28)

7- **The manuscript mentions the use of performance metrics such as memory efficiency and robustness improvement, which are crucial for evaluating the effectiveness of video watermarking systems. However, it does not specify the statistical methods used to analyze these metrics, such as hypothesis testing, confidence intervals, or error analysis.**

**Response**

In the revised version, hypothesis testing (ANOVA analysis) with a confidence interval to estimate the range within which the true population mean normalized correlation (MNC) likely lies was conducted.

ANOVA (Analysis of Variance) analysis can be a powerful tool for comparing the performance of video watermarking systems under different attack scenarios in terms of mean normalized correlation (MNC). In this case, we clearly define the experimental design, including: Independent Variable: Types of attacks (e.g., compression, noise addition, cropping). Dependent Variable: Mean Normalized Correlation (MNC). Control Variables: Factors that may influence MNC but are not the focus of comparison (e.g., watermarking algorithm, video content). After that, we collect MNC values for each attack scenario from multiple trials or samples (*N*=12). We apply a one-way ANOVA to test for significant differences in MNC values among different attack scenarios.

The null hypothesis (H0) is that there is no significant difference in the mean of MNC across attack types.

The alternative hypothesis (H1) is that at least one attack type differs significantly in the mean of MNC.

In this case, the F-statistic indicates whether there are significant differences among group means, and the p-value indicates the probability of observing the data if the null hypothesis were true. The results of a one way ANOVA (F-test) are revealed in Table 3. The results confirm that (1) there are no statistically significant differences between the performances of the videos under different attacks, as the value of F was 0.525, with a significant level greater than 0.05. (2) This could be explained by the fact that all videos are resistant to different types of attacks.

Table 3: The differences between the videos under study when subjected to different types of attacks using one-way ANOVA (F-test).

|  | **Video** | | | ***N*** | **Mean** | | **Std.**  **deviation** | **F** | | **P-value** | | **Result** | |  |
| --- | --- | --- | --- | --- | --- | --- | --- | --- | --- | --- | --- | --- | --- | --- |
| **Attacks** | | **Silent** | 12 | | | 0.999592 | 0.00246 | | 0.525 | | 0.66 | | N.Sig | |
|  |  | **Foreman** | 12 | | | 0.999500 | 0.00022 | |  |  |  |  |  |  |
|  |  | **Mobile** | 12 | | | 0.999567 | 0.00021 | |  |  |  |  |  |  |
|  |  | **Hall monitor** | 12 | | | 0.999492 | 0.00026 | |  |  |  |  |  |  |

Page (33)

8- **The manuscript could benefit from a comparative statistical analysis with existing methods. Including statistical comparisons (e.g., t-tests, ANOVA) could help highlight the improvements the proposed method offers over traditional or other blockchain-based video watermarking approaches.**

**Response**

In the revised version, Table 5 highlights the improvement of the suggested model in comparison with other non-blockchain video watermarking techniques in terms of imperceptibility metrics.  Furthermore, Table 6 compares the suggested blockchain-based video watermarking approach with non-blockchain approaches.

| Aspect | Non-Blockchain technique | Blockchain-Based technique |
| --- | --- | --- |
| Storage | Centralized | Decentralized via IPFS |
| Data Integrity | Managed by central authority | Ensured by blockchain immutability |
| Security | Vulnerable to tampering | High security with cryptographic methods |
| Tamper Resistance | Lower | Higher |
| Transparency | Limited | High due to public ledger |
| Audit Trail | Challenging | Clear and immutable |
| Robustness to Changes | Limited | High with perceptual hashing |
| Single Point of Failure | Yes | No |
| Scalability | Generally scalable | Potential issues with large-scale data |
| Verification | Manual and centralized | Automated and decentralized |

Table 6: comparison between Non-Blockchain technique and Blockchain-Based video watermarking technique

Page (37)
